# Supplementary material for: Sterilization of image steganography using self-supervised convolutional neural network
Source: PeerJ Comput Sci. 2024 Sep 24;10:e2330. doi: 10.7717/peerj-cs.2330 (PMC11623220; doi:10.7717/peerj-cs.2330)
Supplement: Supplemental Information 3 [file peerj-cs-10-2330-s003.pdf]

1.Subject: Request to add Liu Keren author to the manuscript

Dear members of the editorial board, on behalf of the team of authors of this article, I am writing to you to express our request that we would like to add authors to the paper. I will explain the addition of new authors below.

Specifically, in terms of evaluating the generalization ability of the proposed method, she added LSB, a spatial steganography algorithm, to the steganography method, and completed the experiments on the effects of the proposed method in different social media and different types of images. In terms of comparative experiments, she compares the method in this paper with that in the paper "Steganogram removal using multidirectional diffusion in fourier domain while preserving perceptual image quality……"Her participation not only enriches the content of the paper, but also improves the quality of the paper as a whole. She plays a key role in the improvement of supplementary experimental part.

We hope that you will understand our intention to add the author and accept the reasonableness of this decision. If you need more information or have any concerns, please feel free to contact me. We thank you very much for your understanding and support in this matter.

After consultations, all the authors agreed with the addition of authors in this paper， Below are the signatures of all authors. Liu Keren's specific contributions to this research manuscript are as follows:

1.Supplementary comparative experiment

Objective evaluation

In this section, experiments are conducted for steganography algorithms DMAS, GMAS and LSB to verify the effectiveness of our method. The BER of the sanitized images generated by the SS-Net under different payloads and image quality factors is taken as the mean value for

2.The different texture images for the results of our display experiment

above data show the effectiveness of the method in this paper to maintain the quality of images.

In addition, the different texture images for the results of our display experiment are shown in Table 7. As can be seen from Table 7, it can be concluded that on different texture images, the

Metrics	Images with different textures			
	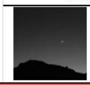	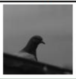	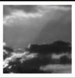	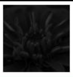
BER	0.59701	0.59633	0.55556	0.52825
PSNR	50.8473	51.3308	44.8439	53.1643
SSIM	0.9957	0.9974	0.9943	0.9964

Table 7

3.Subsequent Supplementary experiments

Comparative experiment

In order to compare with this method, typical and latest steganography sterilization methods are selected in this section: the AO-Net method (Zhu et al. 2021), DFT method (Geetha et al. 2021) and the SC-Net (Zhu et al. 2023) method. Among them, the AO-Net method is the first

Method	Metrics		
	BER	PSNR	SSIM
SC-Net	0.32975	50.34086	0.99615
AO-Ne	0.47122	45.60663	0.98232
DFT	0.47114	46.92	0.9428
Our method	0.4625	49.00888	0.99415

Table 11
